# Co-delivery nanoparticles of anti-cancer drugs for improving chemotherapy efficacy

**DOI:** 10.1080/10717544.2017.1410256

**Published:** 2017-12-01

**Authors:** Shan-Shan Qi, Jia-Hui Sun, Hao-Han Yu, Shu-Qin Yu

**Affiliations:** a Jiangsu Province Key Laboratory for Molecular and Medical Biotechnology, College of Life Sciences, Nanjing Normal University, Nanjing, The People’s Republic of China;; b Cancer Pharmacology Crown Bioscience Inc, Taicang, The People’s Republic of China;; c Nanjing DeBioChem Inc, Nanjing, The People’s Republic of China

**Keywords:** Combination chemotherapy, nano drug co-delivery system (NDCDS), multidrug resistance (MDR), targeting nanoparticles, drug delivery materials

## Abstract

To achieve superior therapeutic efficacy, the combination chemotherapy using two or more anticancer drugs in clinical practice has been generally accepted as a feasible strategy. On account of the concept of combination chemotherapy, co-delivery of anticancer drugs with nanotechnology gradually becomes a desired strategy and one of the research frontiers on modern drug delivery. In recent years, nano drug co-delivery system (NDCDS), which loads at least two anticancer drugs with different physicochemical and pharmacological properties into a combination delivery system, has achieved rapid development. NDCDS synergistically inhibited the growth of the tumor compared with the free drugs. In this review, we highlighted the current state of co-delivery nanoparticles and the most commonly used nanomaterial, discussed challenges and strategies, and prospect future development.

## 1. Introduction

Cancer is a class of diseases characterized by the uncontrolled growth and spread of abnormal cells. There are more than 100 different types of cancer. Cancer can develop from almost any type of cell in the body. In the United States, the American Cancer Society each year estimates the numbers of new cancer cases and deaths that will occur in the current year and compiles the most recent data on cancer incidence, mortality, and survival. The numbers of new cancer cases is more than 1.6 million and caused nearly 0.6 million deaths in 2014 (Siegel et al., 2014); about 1.6 million new cancer cases and 0.59 million cancer deaths are projected to occur in 2016 (Siegel et al., 2016). The cancer death rate has dropped by 23% since 1991. Despite this progress, death rates are increasing for cancers of the liver, pancreas, and uterine corpus (Siegel et al., 2016). It is still the second leading cause of death, exceeded only by heart disease. The three most prevalent cancers are prostate, colon, and rectum, and melanoma among males; and breast, uterine corpus, and colon and rectum among females (Miller et al., 2016). In The People’s Republic of China, with increasing incidence and mortality, cancer became the leading cause of death and a major public health problem. A total of 3.68 million new cancer cases and 2.23 million cancer deaths were estimated in 2013. Cancers of lung, female breast, stomach, liver, colon–rectum, and esophagus were the most common cancers, accounting for about half of all cancer new cases. Lung cancer, liver cancer, stomach cancer, esophageal cancer, and colorectal cancer were the five leading causes of cancer death, accounting for about 60% of all cancer deaths (Chen et al., 2017). In 2015, 4.2 million new cancer cases and 2.8 million cancer deaths occurred (Xu et al., 2016a). Based on World Cancer Report 2014 by World Health Organization (WHO), globally, the cancers diagnosed most common were those of the lung (1.8 million cases, 13.0% of the total), breast (1.7 million, 11.9%), and large bowel (1.4 million, 9.7%) in 2012. The most common types of cancer in males are lung, prostate, colorectum, stomach, and liver cancer; and, in females are breast, colorectum, lung, cervix and stomach cancer. The most common causes of cancer death were lung cancer (1.6 million, 19.4% of the total), liver cancer (0.8 million, 9.1%), and stomach cancer (0.7 million, 8.8%).

Cancer cells contain mutated oncogenes and tumor suppressor genes, which enable them to sustain proliferative signaling, evade growth suppressors, resist cell death, induce angiogenesis, and enable replicative immortality and active invasion and metastasis. The complex signaling pathways in cancer cells, along with the multiple evading mechanisms of programmed cell death, make cancer treatment extremely challenging.

The primary goal of cancer treatment is to cure cancer or to considerably prolong life, as well as to improve the patient’s quality of life. The traditional treatment for cancer includes surgery, chemotherapy and radiation therapy, and many others. Classical chemotherapy is to utilize the cytotoxic chemotherapeutic agents for the treatment of cancer by interfering with the cell division process. However, the effectiveness of chemotherapy is often limited by its toxicity and side effects to normal tissues and organs in the body. Common toxicity and side effects include bone marrow hematopoietic dysfunction, hair loss, nausea, fatigue, and vomiting. These effects are generally related to the mechanism of chemotherapeutic drugs. In addition, the low bioavailability and poor targeting of chemotherapeutic agents are also one of the causes of chemotherapy failure.

In the chemotherapy, invention of innovative compounds and new formulations of existing drugs with modern pharmaceutics technology is two main strategies for the research and development (R&D) of drugs. Innovative drug R&D is the process of bringing a new pharmaceutical drug to market once a lead compound has been identified through the process of drug discovery. It includes pre-clinical research on *in vitro* molecules, cells, tissues, organs, and *in vivo* animals, filing for regulatory status, such as via the United States Food and Drug Administration (FDA) for an investigation new drug to initiate clinical trials on humans, and may include the step of obtaining regulatory approval with a new drug application to market the drug. The R&D project of an innovative drug is characterized by high attrition rates, large capital expenditures, and long timelines. Because advantages and disadvantages of the many first-line chemotherapy drugs were clearly known, R&D of new drug formulations seems to have more advantages over innovative drug.

Combination chemotherapy is a feasible strategy for the combine application of two or more anticancer drugs with different pharmacological mechanisms or with non-overlapping toxicities and side effects (Hu et al., 2010). Based on the idea, nano drug co-delivery system (NDCDS), which loads at least two anticancer drugs with different physicochemical and pharmacological properties into a delivery system, can exert better anticancer effects compared with single-drug loaded systems. In recent years, NDCDS has achieved rapid developments. Some NDCDS had been established to load two small-molecule anticancer drugs (Guo et al., 2014; Lv et al., 2014; Yi et al., 2015), or a small-molecule drug plus a macromolecule anticancer drug (Dai and Tan, 2014; Jiang et al., 2014; Teo et al., 2016; Wang et al., 2016a). NDCDS synergistically inhibited the growth of the tumor compared with free drugs. In this review, we highlighted the current state of NDCDS and the most commonly used drug delivery materials during last five years, discussed challenges and strategies, and prospect future development.

## 2. Combination chemotherapy and NDCDS

### 2.1. The category of anticancer drugs

According to the action mechanism of anticancer drugs, the cancer cells can be targeted at the DNA, RNA or protein on a molecular level, at the organelle or nucleus on cell level, and at endothelium and extracellular matrix on tissue level (Sun et al., 2016b). Most classic chemotherapeutic agents interact with DNA of cancer cells, whereas monoclonal antibodies are directed against proteins or the endothelium and extracellular matrix (Espinosa et al., 2003).

Traditional small molecule chemotherapeutic agents, which can damage DNA and interfere with cellular mitosis, are still the mainstream of cytotoxic anticancer drugs in clinical application. They include alkylating agents, anti-microtubule agents anti-metabolites, topoisomerase inhibitors, cytotoxic antibiotics, etc. Anti-microtubule agents include vinca alkaloids and taxanes. The vinca alkaloids, for example vincristine (VCR) and vinblastine, can prevent the formation of the microtubules, whereas the taxanes, including paclitaxel (PTX) and docetaxel (DTX), can prohibit the microtubule disassembly. As a result, cancer cells do not complete mitosis. Topoisomerases are enzymes that participate in the over-winding or under-winding of DNA. Topoisomerase inhibitor is a type of anticancer drugs that affect the activity of topoisomerase and disrupt catalytic turnover. Topoisomerase I inhibitors, trinotecan and topotecan (TPT), are semi-synthetically derived from camptothecin (CPT), which is obtained from the Chinese ornamental tree *Camptotheca acuminata*. Etoposide and teniposide are Topoisomerase II inhibitors. Etoposide is also a semi-synthetic derivative of podophyllotoxin isolated from the rhizome of the wild mandrake (*Podophyllum peltatum*). Etoposide forms a ternary complex with DNA and the topoisomerase II enzyme, prevents religation of the DNA strands, which make DNA break down. Since cancer cells rely on this enzyme more than that of normal cells, this phenomenon will cause errors in DNA synthesis and promotes apoptosis of the cancer cells. The anticancer drug of platinum-based family is consisted of cisplatin (CDDP), carboplatin, oxaliplatin, and nedaplatin. They are antineoplastic drugs by blocking the duplication of DNA and used for treating a number of cancers, including testicular cancer, ovarian cancer, breast cancer, bladder cancer, and lung cancer. Cytotoxic antibiotics obtained from the bacterium *Streptomyces peucetius*, for example doxorubicin (DOX) and daunorubicin, were the first-line chemotherapeutic drugs. Their anticancer mechanisms include DNA intercalation (molecules insert between the two strands of DNA), generation of highly reactive free radicals that damage intercellular molecules and topoisomerase inhibition. Commonly used anticancer drugs in NDCDS are showed in Table 1.

**Table 1.** The pharmacological characteristics of usual anticancer drugs used in NDCDS.

Category	Name	Pharmacological target	Mechanisms of action	Indications
SMCA	Paclitaxel (PTX)	Microtubules	Promotes the polymerization of tubulin	Ovarian, breast, head and neck, lung cancer
SMCA	Docetaxel (DTX)	Microtubules	Promotes the polymerization of tubulin	Ovarian, breast, head and neck, lung cancer
SMCA	Cisplatin (CDDP)	Nucleus	Interacts with the DNA and forms the inter- or intra-strand chain cross-linking, prevents the replication of DNA	Testicular, ovarian, breast, and bladder cancer
SMCA	Carboplatin	Nucleus	Causes intra- and inter strand DNA crosslinks blocking DNA replication and transcription	Ovarian, lung, head, neck, and brain cancer
SMCA	Oxaliplatin (OXA)	Nucleus	Causes intra- and inter strand DNA crosslinks blocking DNA replication and transcription	
SMCA	Doxorubicin (DOX)	Nucleus	Intercalates into DNA double strand and inhibits the progression of DNA topoisomerase II, stopping replication process	AML, ALL, solid tumors and sarcomas
SMCA	Daunorubicin (DRB)	Nucleus	Inhibits both DNA and RNA synthesis and inhibits DNA synthesis. Topoisomerase II inhibitor	AML, ALL, CML, and Kaposi's sarcoma
SMCA	Camptothecin (CPT)	Nucleus	Binds to the topoisomerase I and DNA complex, prevents DNA re-ligation and causes DNA damage	
SMCA	Topotecan (TPT)	Nucleus	Topotecan's active lactone form intercalates between DNA bases in the topoisomerase-I cleavage complex	
SMCA	Irinotecan (CPT-11)	Nucleus	Topoisomerase-I inhibitor	Colon and lung cancer
SMCA	10-Hydroxycamptothecin(10-HCPT)	Nucleus	Topoisomerase-I inhibitor	
SMCA	SN-38	Nucleus	Is an active metabolite of CPT-11, inhibits DNA topoisomerase I	
SMCA	Etoposide	Nucleus	Forms a complex with DNA and topoisomerase II, causes DNA strands to break	Testicular, lung, and ovarian cancer. Lymphoma
SMCA	Teniposide	Nucleus	Topoisomerase II inhibitor	ALL and lymphoma.
SMCA	Vincristine (VCR)	Microtubules	Binding to the tubulin, stopping the cell from separating its chromosomes during the metaphase	ALL, AML, neuroblastoma, and lung cancer
SMCA	Curcumin (CUR)	Nucleus, P-gp,mitochondria	Multiple cell signaling pathways	
Antibody	Trastuzumab Herceptin	ERBB2	Inhibition of ERBB2 signaling and ADCC	Breast cancer
Antibody	Bevacizumab	VEGF	Inhibition of VEGF signaling	Colon and lung cancer, glioblastoma
Antibody	Cetuximab	EGFR	Inhibition of EGFR signaling and ADCC	Colorectal, lung, and head and neck cancer

With the developments of modern biotechnology, antibodies and genes have made great progress in anticancer therapy (Scott et al., 2012). Over the past few decades, benefited from the unique targeting capabilities, antibodies have emerged as effective anti-cancer agents and attracted enormous interests in pre-clinical and clinical applications. Currently, FDA approved monoclonal antibodies in oncology and their mechanisms of action are also showed in Table 1 (Zhang et al., 2007). Gene therapy against cancer are usually designed for the delivery of nucleic acids (e.g. DNA, siRNA, shRNA, antisense oligonucleotides) to express pro-apoptotic proteins, substitute mutated genes, or up-regulate cytotoxic immune cytokines, as well as silence unwanted gene expression by RNA interference (Mujokoro et al., 2016).

### 2.2. Combination chemotherapy

To date, chemotherapy, together with surgery and radiotherapy, is still used as three most effective modalities suppressing tumor cell growth and invasion in clinics. Compared with both surgical resection and radiation therapy, the chemotherapeutic drugs possess several advantages, such as less invasion, administration convenience, and age-independent efficacy. Unfortunately, despite all these advantages, the efficacy of most chemotherapeutic agents is hampered by poor pharmacokinetics and non-specific distribution. Moreover, using single chemotherapeutic drug often brings several issues to anticancer treatment, such as unfavorable toxicity, development of drug resistance, and limited regime in clinical uses. For the purpose to overcome the therapeutic limitations of single drug chemotherapy, multifunctional nano-platform for co-delivery has been proposed as an impactful solution to improve chemotherapy efficacy against cancer.

The chemotherapy can observably reduce the mortality rate of various malignant tumors and is still one of the most important therapeutic strategies. However, usual single drug chemotherapy may cause serious toxicity-adverse effects, lose anticancer sensitivity and engender multidrug resistance (MDR) (Gottesman et al., 2002). Various reasons for the emergence of MDR include that the cancer cell lessens intracellular drug concentration induced by decreasing the uptake and increasing the efflux associated with P-glycoprotein (P-gp) overexpression, alters the target or metabolic pathway of the drug, activates DNA repair function, and so on.

MDR in cancer cells is also a challenge to improve the effect of chemotherapy. MDR is another important factor in the failure of many forms of chemotherapy. MDR is characterized by a decreased sensitivity of tumor cells not only to the particular drug employed for chemotherapy but also to a broad spectrum of drugs with neither obvious structural similarities nor common targets. It is generally recognized that MDR is associated with the overexpression of P-gp in cancer cells. P-gp is an ATP-dependent membrane transport protein with an efflux-related function for intracellular exogenous compounds, such as drugs and xenobiotics (Borst et al., 2000). Numerous studies have demonstrated that P-gp plays a significant role in MDR of cancer chemotherapy. Notably, these small molecule anticancer drugs mostly belong to P-gp substrate. Owing to MDR, single-drug chemotherapy may need the clinical treatment scheme with higher dose and longer course, which eventually leads to intolerable toxicity. Therefore, single chemotherapy cannot completely eradicate cancers.

In order to achieve superior therapeutic efficacy or to minimize the adverse effects on the normal organs, and to prevent the development of MDR, combination chemotherapy is a feasible strategy with the combined application of two or more anticancer drugs with different pharmacological mechanisms or non-overlapping toxicities (Hu et al., 2010).

In 1965, based on the fact that some cancers could produce resistant to single chemotherapy, Holland et al. simultaneously used four anticancer drugs, methotrexate, VCR, 6-mercaptopurine (6-MP), and prednisone, to treat acute lymphoblastic leukemia (ALL) in children. This therapeutic method was considered to be combination chemotherapy for cancers at first time (Schilsky et al., 2006). Devita et al. reported that 43 patients with Hodgkin's disease were treated with a combination of VCR, nitrogen mustard (or cyclophosphamide), procarbazine and prednisone for 6 months (Devita et al., 1970). A randomized phase III study of DOX versus CDDP/interferon α-2β/DOX/fluorouracil (PIAF) combination chemotherapy for hepatocellular carcinoma had a higher overall response rate and better survival than that of single chemotherapy with DOX (Yeo et al., 2005). Combination chemotherapy improves response rate and failure-free survival compared with single chemotherapy, but there was not any statistically significant difference in the primary end point of overall survival (Lilenbaum et al., 2005). Currently, combination chemotherapy is almost the standard treatment program for most advanced cancers in clinical treatment.

The combination index (CI) is usually used to describe the advantage of combination chemotherapy (Chou, 2010). The inhibitory concentration (IC_50_) values and the CI are calculated according to following equation (Chou, 2010):

$$\text{CI=}\frac{{\text{D}}_{\text{A}}}{{\text{D}}_{\text{X.}\text{A}}}+\frac{{\text{D}}_{\text{B}}}{{\text{D}}_{\text{X.}\text{B}}}$$

where *D*_θ_ and *D*_x_, *θ* represents the concentration of drugs used in combination to achieve *x*% effectiveness and concentrations of single drug to achieve *x*% drug effect, respectively. The CI was mathematical indicator for drug synergy, which is more accurate than a simple efficacy summation. Combination chemotherapy can result in synergistic, additive, or antagonistic interaction effects at different concentration ratios.

### 2.3. Nano drug co-delivery system (NDCDS)

On account of the concept of combination chemotherapy, co-delivery of anticancer drugs with nanotechnology gradually becomes a desired strategy (Teo et al., 2016). Over the last two decades, NDCDS has got the rapid development in cancer chemotherapy. NDCDS is recognized to have the advantages of breaking through the biological barrier, improving the target property, and enhancing penetration ability on the tumor tissue.

NDCDS, which loads at least two anticancer drugs with different physicochemical and pharmacological properties into a delivery system, is designed for the purpose of clinical combination chemotherapy. The combination of chemotherapeutic agents and other drugs that target different cellular pathway, such as chemosensitizers, can delay the cancer adaptation; consequently reduce the cancer cell mutation possible. In recent decades, NDCDS has emerged as a promising strategy of combined anticancer therapy to combat sophisticated cancer pathway for better therapeutic efficacy. In addition, it can get higher targeting selectivity.

Although NDCDS is relied on the concept of combination chemotherapy, there are slight differences between each other. For example, in clinical practice, because the drugs administered for combination chemotherapy are rarely mixed in the same infusion bottle, the pharmaceutical incompatibility of drugs associated with physicochemical properties is not usually taken into account. In this condition, there is less influence on the pharmacokinetic characteristics of the drugs (Ana Catarina Pinto and Simões, 2011). However, NDCDS makes drugs enter the blood circulation at the same time. Hence, it is a feasible approach for cancer chemotherapy to use excellent NDCDS with a sequential and precision release characteristic to transport anticancer drugs to their pharmacological targets (He et al., 2015; Kemp et al., 2015).

## 3. The model drugs in NDCDS

### 3.1. Co-delivery of different small molecule chemotherapeutic drugs

DOX and PTX are most commonly used in NDCDS chemotherapeutic drugs, which have different pharmacological mechanisms and different subcellular targets (Wang et al., 2011; Duong & Yung, 2013; Feng et al., 2014; Lv et al., 2014; Yu et al., 2014; Chen et al., 2015). The co-delivery polyethylene glycol-polylactic-*co*-glycolic acid (PEG-PLGA) nanoparticles-loaded DOX and PTX could enhance anti-tumor efficacy of the combination chemotherapy with free DOX and PTX on non-small cell lung cancer (Wang et al., 2011; Lv et al., 2014). Feng et al. designed and synthesized a co-delivery system of DOX and PTX for inhalation treatment of pulmonary tumor. The results indicated that the combination of free DOX and free PTX at a molar ratio of 5/1 showed the best synergistic therapeutic effect. However, the drugs exhibited more synergism in the NDCDS at a molar ratio of 2/1, due to the difference in drug release rate (Feng et al., 2014). Wang et al. developed core-shell methoxy PEG-PLGA nanoparticles loaded with DOX and PTX. The nanoparticles showed a synergistic effect due to better efficiency growth suppression on the cancer cells compared with the single delivery of either DOX or PTX at the same concentrations (Wang et al., 2011).

Cisplatin (CDDP) is an anti-neoplastic drug, which can block activity on DNA duplication and belong to the platinum-based family of medications. Some NDCDS of CDDP plus PTX, or DTX, or rapamycin were studied to get a synergistic efficacy for ovarian cancer and melanoma (Cai et al., 2014; Guo et al., 2014; Song et al., 2014b). Similar synergistic effects were got in the evaluation on co-delivery of daunomycin and oxaliplatin by biodegradable polymers for enhanced anticancer therapy (Xiao et al., 2012a). The other co-delivery of small molecule chemotherapeutic drugs, for instance, TPT and vincristine (Zucker et al., 2012), irinotecan and DOX (Wang et al., 2015a), 10-hydroxycamptothecin with DOX (Zhang et al., 2013), can be found in some studies for enhancing anticancer efficacy utilizing combination chemotherapy.

NDCDS can also effectively deliver two therapeutic agents with different chemical properties, such as hydrophobicity plus hydrophilicity drugs (Singh et al., 2011). An example is the co-encapsulation of DOX and mitomycin C within polymer-lipid hybrid nanoparticles (DMPLN) to achieve tumor-targeting delivery in a murine breast tumor model. Compared with free drug cocktails, DMPLN showed higher levels of cancer cell apoptosis and reduced organ toxicity (Zhang et al., 2016b). Similar synergistic effect also can be found in Ma’s report in which a redox-responsive hydrogel with controlled release was designed for co-encapsulation 5-fluorouracil (5-FU) and CPT (Ma et al., 2013).

In our research group, Wang et al. designed and prepared a kind of NDDS, which is a novel nanoparticle with several smaller nanoparticles located at a larger nanoparticle, named S-D1@L-D2 NPs for single delivery or co-delivery with small-molecule anticancer drugs (Wang et al., 2016c). In other study, we prepared chitosan-alginate nanoparticles carrying DOX (CS-ALG-DOX NPs) with a smaller diameter of about 20 nm formed S-D1 NPs; vitamin E d-a-tocopheryl polyethylene glycol 1000 succinate-modified PLGA nanoparticles carrying VCR (TPGS-PLGA-VCR NPs) with a larger diameter of about 200 nm constituted L-D2 NPs. Some CS-ALG-DOX NPs loaded into TPGS-PLGA-VCR NPs formed CS-ALG-DOX@TPGS-PLGA-VCR NPs (or S-D1@L-D2 NPs). After CS-ALG-DOX@TPGS-PLGA-VCR NPs having entered A549/Taxol cell, VCR and the inner nanoparticles were released at pH 5.8. The drug VCR would target microtubule in the cytoplasm, and CS-ALG-DOX NPs would enter the nucleus of MDR cell through the nuclear pores. The inner nanoparticles could release DOX at the nucleus to target DNA in intra-nuclear alkaline environment (pH = 7.4). These results substantiated that S-D1@L-D2 NPs was a co-delivery system of intracellular accurate release of loaded-drugs with pH sensitive characteristics. S-D1@L-D2 NPs obviously enhanced *in vitro* cytotoxicity and the *in vivo* anticancer effects of the co-delivery drugs, meanwhile reduced their adverse effects. The S-D1@L-D2 NPs can be considered as an innovative platform for the co-delivery of drugs in combination chemotherapy at the clinical treatment of MDR tumor (Zhang et al., 2017a). In NDCDS, this separate carriers system has the advantage of intracellular accurate drug release.

### 3.2. Co-delivery of small molecule chemotherapeutic drugs plus antibody

Nowadays, numerous combinations of chemotherapeutic agents with monoclonal antibody are being applied for the treatment of various cancers. In the UK, for example, CPT-11/cetuximab is being used to treat breast, pancreatic, and bowel cancers. Although the development for the nano-formulation of this combined therapy has not met the demand of market, NDCDS of small molecule chemotherapeutic drugs plus antibody have become a hot spot of research owing to reduction of cumulative toxicity of their native form on normal cells and improvement of their anti-cancer effects. The design concept of co-delivery of small molecule chemotherapeutic drugs plus antibody is based on the fact that drug resistance to some chemotherapeutic drugs is the main obstacle for efficient treatment in cancer patients. Owing to overexpressing of some proteins, for example epidermal growth factor receptor (EGFR), in cancer cells with drug resistance, specific monoclonal antibody-modified nanoparticles can be used to overcome EGFR resistance cancer.

Herceptin (Trastuzumab), a monoclonal antibody anticancer drug, can recognize HER2/neu and is often used in combination with small-molecule chemotherapeutic drugs in the treatment of metastatic breast cancer. Several research groups had reported research on co-delivery of DOX, CDDP, or PTX with Herceptin (Lee et al., 2009; Liu & Feng, 2011; Mi et al., 2013). NDCDS with pH sensitiveness can enhance cancer therapy efficacy with the simultaneous encapsulation of hydrophilic and hydrophobic compounds (Chiang et al., 2014). Lee et al. reported cationic micelles assembled from copolymer, P(MDS-co-CES), to co-deliver PTX and Herceptin to achieve targeting delivery of PTX to HER2/neu-overexpressing human breast cancer cells. In micelle nanoparticles, herceptin was capped onto the surface of the nanoparticles, which is facilitated by hydrogen bonding and/or hydrophobic interaction between the antibody and the nanoparticles. The NDCDS of Herceptin and PTX showed significantly higher cytotoxicity compared with free PTX in different cancer cell lines. It is important to note that the enhancement of anti-cancer effects displayed a dependence on HER2-overexpressing level (Lee et al., 2009).

Another monoclonal antibody drug was also used to co-delivery small molecule chemotherapeutic drugs, for example anti-VEGFR-2 antibody and irinotecan (Sharma et al., 2014a), cetuximab and DOX or gefitinib (Wang et al., 2016b). Wang et al. designed the cetuximab-modified mesoporous silica nanoparticles (MSNs) as the drug carrier to specifically target EGFR-mutant lung cancer cells and efficiently release loaded drugs DOX and gefitinib. Their results demonstrated that the co-delivery nanoparticles could overcome EGFR-TKI resistance and held strong potential for effective management of EGFR-mutant lung cancer (Wang et al., 2016b).

### 3.3 Co-delivery of chemotherapeutic drugs plus genes

Due to the aforementioned complexity of signal pathway in cancer cells, chemotherapeutic agents have their own limitations in completely eradicating cancer cells (Mujokoro et al., 2016). With a deeper understanding of the genetic aberrations involved in cancer cell signaling, combined therapy of genes with chemotherapeutics was prompted to regulate those critical pathways by evading chemical interference. Thus, a combination of gene therapy and chemotherapy can gain efficacy by interfering with multiple cancer pathways simultaneously.

Supplementing down-regulation or replacing mutated genes by delivering therapeutic plasmid DNA (pDNA) is an important model in which gene therapy takes action. p53 is the most frequently mutated tumor-suppressor gene in human cancers. As a tumor-suppressor gene, *p53* is involved in a process of programmed cell death, which is known as apoptosis. It is reported by many researchers that the loss of p53 function in cells with mutated p53 genes induces several oncogenic properties such as increased genomic instability and cell proliferation. Zhang et al. fabricated DSPE-PEG-AA(1,2-distearoryl-*sn*-glycero-3-phosphoethanolamine-*N*-[methoxy(polyethyleneglycol-2000)] ammonium salt-anisamide) modified high-density lipoprotein-based nanoparticles (DSPE-PEG-AA/rHDL) for co-encapsulation of dichloroacetate (DCA) and p53 together for potential cancer treatment (Zhang et al., 2016a). *In vitro* study in human lung adenocarcinoma cell line A549 confirmed efficient cytoplasmic drug delivery and gene transfection mediated by scavenger receptor class B type I (SR-BI) and sigma receptor dual-targeting function. Further *in vivo* investigation on nude mice bearing A549 tumor xenografts revealed that the complexes of co-formulation possessed specific tumor targeting and strong antitumor activity. Similarly, Xu et al. fabricated double-wall microspheres consisting of a PLGA core surrounded by a PLA shell for co-encapsulation of DOX and gene encoding the p53 tumor suppressor protein (chi-p53), resulting in significantly enhanced cytotoxicity and increased activation of caspase 3 in HepG2 cells compared to those treated with free drugs (Xu et al., 2012, 2013).

Another approach to achieve synergistic effect in treating cancer using gene and chemotherapy is co-delivery of various chemotherapeutic agents and RNAs. A polyamidoamne dendrimer functionalized graphene oxide (GO-PAMAM) was designed in order to achieve synergistic effective of DOX and MMP-9 shRNA plasmid in breast cancer. It achieved significantly better transfection efficiency and biocompatibility compared with PEI-25k, as well as significantly inhibited MMP-9 protein expression in MCF-7 cells. Moreover, the effect of DOX and MMP-9 shRNA plasmid co-delivery was more significant than that of the single drug (Sharma et al., 2014b). Jia et al. developed a flexible controllable nanoplatform consisting of phenylboronic acid-tethered hyperbranched oligoethylenimine (OEI600-PBA) and 1,3-diol-rich hyperbranched polyglycerol (HBPO) via pH-dependent self-assembly for combination of chemotherapy with RNA interfering (RNAi) therapy. Beclin1 siRNA, which can suppress the post-chemotherapy survival, is introduced as therapeutic model drug to evaluate the antitumor capability of NDCDS, as well as DOX. As a result, the nano-assembly-mediated combinational treatment displayed even better *in vitro* anticancer effects and effective *Beclin1* gene silence than free DOX, accompanied with DOX-induced autophagy suppress and higher sensitivity to DOX chemotherapy (Jia et al., 2015).

During recent years, there has been a remarkable progress in NDCDS for combination therapy of genes and chemotherapeutic agents. Disappointingly, until now there are no FDA approved gene anticancer therapy or gene-chemo combination therapy products on the market. Therefore, it still needs further studies for clinical application of nanotechnology in co-formulation of genes and chemotherapeutic agents (Weissig et al., 2014).

### 3.4 Co-delivery of photo-thermal therapy drug

Multi-modal therapies have the advantages of eliminating cancer cells through inescapable and infallible processes. Upon receipt of an external trigger (e.g. laser, NIR, X-ray, and magnetic field), physically activated modalities (e.g. photo-thermal, photodynamic, radio-, and magnetically assisted therapies) also offer a synergistic approach to destroy cancer cells via generating a wide variety of therapeutic effects. In photo-thermal therapy, drug delivery materials with a high absorption cross-section serve to eliminate cancer cells by converting energy from external source (e.g. light, ultrasound, and magnetic field) into heat, which subsequently lead to minimal invasion and uniform hyperthermia. Gold nanomaterial has hyper-thermal effects when triggered by non-invasive deep-penetrating near-infrared light (NIR) exposure, and high biocompatibility and facile surface functionalization. Due to all the superior physiochemical characters and photo-thermal properties, gold nano-biomaterials have been extensively explored as efficient agents for photo-thermal therapy. Chauhan et al. reported gold nanoparticles (AuNPs) composite-Folic acid (FA) conjugated graphene oxide (FA-GO@Au) nano-platforms for chemo-thermal cancer therapy to ablate hepatocellular carcinomas (Chauhan et al., 2016). FA decorated on GO surface was used as functional moiety for active tumor targeting of model drug DOX. After localized NIR irradiation, the release of both DOX and ionic gold from the nanohybrid surface to the cellular vicinity were aggravated, which led to significantly enhanced toxicity against cancer cell. Further mechanistic evaluation demonstrated that *in vitro* G0/G1 phase arrest and early apoptosis provoke were resulted from photo-thermal effects. *In vivo* studies in Balb/c mice model revealed substantial tumor regression and better tumor management upon NIR exposure. Another example of multifunctional gold nanomaterial system with NIR-triggered drug-releasing properties is the core-shell structured DTX-loaded PLGA@Au nanoparticles for tumor-targeted chemo-photo-thermal therapy (Hao et al., 2015). Compared with DTX alone, this novel ANG/GS/PLGA/DTX NPs system afforded much higher anti-tumor efficiency without obvious toxic effects.

Photodynamic therapy is an emerging clinical treatment strategy for malignant cancer with minimal invasion and reduced side effects. When activated by light with proper wavelength, the photosensitizing chemical generates reactive oxygen species via energy transferring, subsequently eliciting cell death and tissue destruction. Combination with chemotherapy can maximize the therapeutic effect of photodynamic therapy. Yao et al. fabricated a di-functional metallo-supramolecular nanogel (SNG) loaded with Zn-Por and DOX for synergistic chemo-photodynamic therapy. This novel co-delivery system can respond to tumor acid microenvironment to release the photosensitizer and anticancer drug to eliminate the lesion cells. In both *in vitro* and *in vivo* studies, DOX-loaded SNG with irradiation exhibited enhanced anticancer therapeutic efficiency, compared with single chemotherapy of DOX or photodynamic therapy using Zn-Por-encapsulated SNG (Yao et al., 2015). Similar synergistically higher anticancer effect can be found in a recent study by Chen et al. (2016) of tirapazamine (TPZ) and paramagnetic Gd^3+^ co-loaded MSNs.

### 3.5. Co-delivery of small molecule chemotherapeutic drug and chemosensitizer

There are increasing investigations on co-delivery of chemotherapy drugs and chemotherapy sensitizers in cancer treatment in recent years. A chemosensitizer is a drug, which does not have a direct cytotoxic effect on cancer cells; it means using small dose of the substance combined with chemotherapy drugs can significantly improve the effectiveness of chemotherapy (Limtrakul, 2007). The chemosensitizers include MDR reverser (or P-gp inhibitor) and autophagy inhibitor. The NDCDS is capable of responsive degradating at the intracellular level and subsequently co-releasing the encapsulated chemosensitizer and anticancer drug to achieve a synergistic effect of chemotherapy.

#### 3.5.1. Co-delivery of small molecule chemotherapeutic drug and P-gp inhibitors

The development of resistance mechanism against anti-cancer agents in cancer cells is a major problem in the chemotherapeutic cancer treatment after repeated administrations. Scientists already found out there are several types of drug resistance: reduced cellular drug concentration by efflux pumps, reduced cellular uptake by membrane lipids alterations, increased or altered drug targets, metabolic alteration of the drug, apoptosis inhibition, the damaged DNA repair, and alteration of the cell cycle checkpoints. Among them, the existence of an array of trans-membrane efflux pumps is probably the chief one. Drug efflux pumps can pump out chemotherapeutic drugs from cells to prevent the cell from following occurrence of apoptosis. A notorious membrane-bound efflux pump commonly over-expressed on most MDR cancers cells is P-gp, which is also known as ABCB1 or MDR1 protein.

In order to enhance the anticancer effect of P-gp substrates, inhibition of the P-gp efflux pump on MDR cells is a smart tactic (Qin et al., 2014). Thus, co-administration of a chemotherapeutic agent with P-gp inhibitors, including verapamil (VER), cyclosporine A, tariquida, and valspodar (PSC833), is used as a promising approach for treating MDR cancer (Bajelan et al., 2012; Sriraman et al., 2015).

VER is a common P-gp inhibitor in drug efflux research. It can combine competitively with the substrate of P-gp on the drug-binding site, and enhances intracellular drug accumulation by decreasing the P-gp efflux. The model drugs in NDCDS co-loaded with verapamil were DOX (Li et al., 2015a; Tang et al., 2015) and DTX (Guo et al., 2017). However, verapamil is a classical Ca^++^ channel blocker used normally for the treatment of cardiac arrhythmia and hypertension. Usually, the doses of verapamil used as a P-gp inhibitor are much higher than those for anti-arrhythmic indications. In consideration of adverse effect induced by pharmacological effect of verapamil, some novel generation P-gp inhibitors without other biological effects were used in co-delivery with anticancer drugs. Tariquidar (XR9576) and elacridar (GG918) are the third generation P-gp inhibitors with potent, specific, and non-competitive inhibition (Sarisozen et al., 2012). Tariquidar can inhibit the ATPase activity of P-gp and be used for increasing toxicity of chemotherapy (Fox & Bates, 2007). Patel et al. had investigated the co-delivery of ariquidar and PTX into tumor cells to reverse the MDR using long-circulating liposomes. Tariquidar- and PTX-loaded long-circulating liposomes showed significant re-sensitization of the resistant variant for paclitaxel, which could be correlated with an increased accumulation of PTX in tumor cells (Patel et al., 2011). Can et al. had developed PEG-PE-based long-circulating micelles co-loaded with elacridar and PTX, and investigated their ability to overcome PTX resistance in two cancer cell lines. Elacridar/PTX-co-loaded micelles were demonstrated to have the highest cytotoxicity compared with both free and micelle PTX, as well as to overcome MDR (Sarisozen et al., 2012).

Curcumin (CUR), a natural hydrophobicphenolic compound derived from the common food spicerhizome of *Curcuma longa* (*turmeric*), has a wide pharmacological activities, including antibacterial, antidepressant, anti-inflammation, antioxidant, and antitumor. It can affect multiple targets and interfere with cell signaling pathways, for example, inducing apoptosis, inhibiting cell proliferation and suppressing inflammation (Xie et al., 2011). It can be used as an anti-cancer drug and a P-gp inhibitor. A glycyrrhetinic acid-modified chitosan-cystamine-poly(ε-caprolactone) copolymer (PCL-SS-CTS-GA) micelle co-delivering DOX and CUR was designed, prepared, and characterized. The PCL-SS-CTS-GA micelles exhibited a character of redox-responsive drug release and GA/pH-mediated endocytosis *in vitro*. Meanwhile, it also facilitated significantly higher cell uptake and strong synergic effect compare to the two drugs (Yan et al., 2016). Co-formulation nanoparticles of DOX and CUR also synergistically improved anti-tumor efficacy in MDR K562 cells *in vitro* and breast carcinoma *in vivo* (Misra & Sahoo, 2011; Sun et al., 2014; Wang et al., 2015b; Yan et al., 2017; Zhang et al., 2017b). The co-delivery micelles of PTX and CUR can overcome MDR in ovarian cancer model (Abouzeid et al., 2014b). Transferrin-targeted nanoparticles co-loaded with CUR and PTX could also efficiently kill MDR ovarian cancer in 3D spheroids and *in vivo* animal model (Abouzeid et al., 2014a; Sarisozen et al., 2014).

P-gp is encoded by the MDR-1 gene, for which siRNA against MDR-1 also can be utilized to knock down protein expression for proteins to achieve MDR inhibition, and subsequently enhance the accumulation of anticancer drugs in the cancer cell for effective anticancer action (Creixell & Peppas, 2012). Sou et al. synthesized a comb-like amphiphilic copolymer methoxypolyethylene glycol-*graft*-poly(l-lysine)-*block*-poly(l-phenylalanine) (mPEG-*g*-PLL-*b*-Phe) to simultaneously transport DOX and P-gp siRNA to MCF-7 cells. DOX/P-gp siRNA-loaded nanomicelles exhibited a preferable release in acidic conditions, efficient internalization, and much higher *in vitro* cytotoxicity compared with DOX-loaded nanomicelles (Suo et al., 2016). In another recent study, He et al. prepared nano metal-organic frameworks (NMOFs) for the co-delivery of CDDP prodrug and MDR gene-silencing siRNAs (Bcl-2), respectively, in ovarian cancer treatment. NMOFs protect siRNA from nuclease degradation, enhance siRNA cellar uptake, and promote siRNA escape from endosomes to silence MDR genes in CDDP-resistant ovarian cancer cells. The delivery vesicles result in a significant enhancement of *in vitro* chemotherapeutic efficacy (He et al., 2014).

#### 3.5.2. *Co-delivery of small molecule chemotherapeutic drug and autophagy inhibitor*

Autophagy is a cellular process that the substrate for degradation been packed into a bilayer membrane structure and transported to lysosome for further degradation. In this process, first, the bilayer membrane abscised from non-ribosomal attachment region of rough endoplasmic reticulum encapsulates part of cytoplasm and organelles, protein, and other components for degradation to form autophagosome. Then the autophagosome fuses with lysosomes to form autolysosome. Finally, the encapsulated contents are degraded by lysosome. The cell will activate autophagy when it is under the stress state, such as nutrition deficiency, lack of growth factors, and hypoxia. Autophagy is an important way for the cell to maintain its own internal environment stable to carry out normal proliferation and differentiation, meanwhile it also can help the cell survive from the stress state (Kondo et al., 2005).

Autophagy can be used as a potential intervention target for cancer therapy. It is generally believed that autophagy is a mechanism of cell survival. In some cases it can selectively remove damaged or superfluous peroxisomes, endoplasmic reticulum, mitochondria, and DNA, and reduce the abnormal protein and organelle accumulation, maintain cell homeostasis (Jin & White, 2007). When cancer cells encounter toxic chemicals or radiation, they will produce a lot of damaged organelles, damaged proteins, and other harmful ingredients. High activity of autophagy can remove harmful substances and inhibit the triggering of death pathway in time, provide emergency substrate and energy, win time, and conditions for the repair of damaged DNA. So some researchers believe that the induction of autophagy is one of the causes of chemotherapy drug resistance in cancer cells. When cancer cells were exposed to chemotherapy drugs, the autophagy level in cells can increase obviously, which is an adaptive response of cancer cells to escape chemotherapy stimulation. It can be concluded that autophagy can cause tolerance to chemotherapy drugs of cancer cells, so inhibition of autophagy will increase the sensitivity of cancer cells to chemotherapeutic drugs. Indeed, the combination therapy regimen with cytotoxic chemotherapy drugs and autophagy inhibitors has been extensively applied in clinical treatment (Wang et al., 2016a).

The research of anticancer drugs combined with autophagy inhibitors has received attention in recent years. Some studies investigated the cytotoxicity on the cancer cell lines of nanoparticles loaded with anticancer drugs and free autophagy inhibitors (Zhang et al., 2014a,b). Zhang et al. prepared DTX-loaded nanoparticles for the treatment of human breast cancer combined with free CQ. The anticancer effect of the co-delivery system was evaluated *in vivo* and *in vitro*. The IC_50_ values revealed that inhibition of autophagy by CQ could boost the *in vitro* therapeutic effects of the H40-PLA NPs by 7.0-fold. The survival rate of DTX-H40-PLA NPs and CQ treated MCF-7 cells decreased apparently compared with the DTX-H40-PLA NPs-treated group. *In vivo* results showed that in the co-delivery group, the weight of cancer bearing mice was significant lower than DTX-H40-PLA NPs treated group, and the cancer volume decreased more (Zhang et al., 2014b). Another study from the same group showed that when treated by different anticancer drugs, such as DOX, DTX, 5-fluorouracil (5-FU), or Tamoxifen, autophagy would all occur in cells. In this study, MCF-7 cells were treated with drug loaded PLAG micelles modified by PEG that were about 40 nm combined with autophagy inhibitor CQ. The result showed that anticancer effect with CQ could be improved significantly with a 12-fold more efficient (Zhang et al., 2014c).

The above researches show that the co-delivery of anticancer drugs and autophagy inhibitors has achieved satisfactory results in both *in vitro* and *in vivo* studies. However, in the above studies, autophagy inhibitors are all free drugs, not loaded in nanoparticles with anticancer drugs, which may lead to side effect of autophagy inhibitors or unknown adverse reactions to normal tissues of the body. CQ may cause anorexia, diarrhea, neutropenia, arrhythmia, etc.

In another study, Sun et al. co-delivered anticancer drugs and autophagy inhibitors for the treatment of breast cancer stem cells. In this study, CQ combined with DOX and DTX to evaluate the anticancer effects of each group. Compared with single treatment, the combined delivery systems NP_CQ_/NP_DOX_ and NP_CQ/DOX_ (NP_CQ_/NP_DTXL_ and NP_CQ/DTXL_) showed most effective and persistent cancer growth inhibitory effect. As for the therapeutic effect, the result demonstrated that encapsulated anticancer drug and autophagy inhibitor into one delivery system is slightly stronger than putting them into two delivery systems (Sun et al., 2016a). In this work, the design of experiments is more comprehensive, and the setting of groups is more systematic. Besides, the autophagy inhibitor is loaded in the nanoparticles, which can avoid the possible adverse reactions.

Shi et al. prepared TPGS-based micelles co-encapsulated with DTX and CQ for enhancing anticancer effects. *In vitro* cytotoxicity of DTX/CQ at ratio of 0.8/0.2 is found to have the strongest synergism effect. The IC_50_ value is 194.74-fold lower than that of free DTX on MCF-7/ADR cells (Shi et al., 2015). Polyphosphazene vesicles for co-delivery of DOX and CQ also enhanced anticancer efficacy for reversal of MDR (Xu et al., 2016b). Co-encapsulating PTX with CQ was designed for treating PTX-resistant carcinoma. As demonstrated by *in vitro* cytotoxicity and apoptosis assay, real-time observation of cellular uptake, along with *in vivo* tissue distribution study, the co-encapsulation of PTX and CQ was validated superior to the mixture of PTX liposome plus CQ liposome due to the simultaneous delivery and synergetic effect of the two drugs (Gao et al., 2015). Zhao et al. prepared chitosan nanoparticles capable of entrapping gefitinib and CQ for multiple drugs combinational therapy. Through western blot analysis, the results showed gefitinib could promote LC3 expression, a marker of autophagosomes. Moreover, CQ in these nanoparticles could overcome autophagy in the resistant cells (Zhao et al., 2015).

#### 3.5.3. *Co-delivery of small molecule chemotherapeutic drug and nitric oxide (NO)*

NO is a molecule with extensive and intricate physiological functions, which depend on the source, concentration, latency, cell type, and phenotype. NO has attracted much attention for its antitumor activity. However, following an inhibition of NO production, some studies find a protection, while others find an exacerbation of tumorigenesis (Hofseth et al., 2003). A novel approach based on modifying gene products that regulate resistance to apoptosis involves NO as a novel therapeutic to overcome MDR when used in combination with or without other therapeutics (Bonavida et al., 2006).

However, owing to NO be gaseous molecules of chemical instability with short half-life, delivering NO directly to cancer tissue is challenging. Song et al. synthesized a NO releasing polymer, nitrate functionalized d-α-tocopheryl polyethylene glycol succinate (TNO3). TNO3 was able to accumulate in tumors and release about 90% of NO content in cancer cells for 96 h. Co-delivering TNO_3_ with DOX to hepatocarcinoma HepG2 cancer cells strengthened the cellular uptake of DOX and enabled the synergistic effect between NO and DOX. Moreover, for DOX-based chemotherapy in tumor-bearing mice, co-administration with TNO3 significantly extended the blood circulation time of DOX and enhanced antitumor efficacy (Song et al., 2014a). Wang et al reported another S-nitroso polysilsesquioxane NPs that possessed simultaneous NO-donating and DOX-releasing properties (Wang et al., 2015c). In order to localize NO generation in solid tumors, Sirova et al. designed polymer carriers based on N-(2-hydroxypropyl) methacrylamide (HPMA) copolymers with incorporated organic nitrates as NO donors. *In vivo* animal test, the co-administration of the polymer NO donor and HPMA copolymer-bound DOX resulted in an improvement in the treatment of murine EL4 T-cell lymphoma (Sirova et al., 2017). To achieve *in situ* release of angiogenic and anticancer drugs in cancer tissue, Yin et al. designed a precise polymeric hybrid micelle system for co-delivering NO and PTX. The hybrid micelles could accumulate in cancer tissue to achieve the synergistic antitumor effect of NO and PTX through modified tumor microenvironment and overcome MDR (Yin et al., 2017).

Overall, there has been increasing evidence for the potential function of NO to overcome MDR. Kim et al. (2017) reviewed recent progress on the mechanism of NO in overcoming MDR and the co-delivery NO and antitumor drugs.

## 4. The drug delivery materials for NDCDS

The biggest challenge in loaded different kinds of anti-cancer agents NDCDS is to find the ideal carrier. Vast arrays of materials, ranging from natural to synthetic and to hybrid, have been developed as co-delivery vesicles for anti-cancer treatment. This object of this section is to review some materials which are most studied for co-delivery in anti-cancer therapy (Table 2).

**Table 2.** Some representative NDCDS: model drugs and biomaterials.

Biomaterial	Model drug 1	Model drug 2	Indications	Ref.
mPEG-PLGA	DOX	PTX	Non-small lung cancer, melanoma, hematoma	Wang et al. (2011)
PCL-SS-CTS-GA micelle	DOX	CUM	Hematoma carcinoma	Yan et al. (2016)
Polymer-lipid hybrid	DOX	Mitomycin C	Murine breast cancer	Zhang et al. (2016b)
Pluronic F-127 diacrylate macromer	5-fluorouracil	Camptothecin		Ma et al. (2013)
P(MDS-*co*-CES) micelle	PTX	Herceptin	Breast cancer	Lee et al. (2009)
DSPE-PEG-AA/rHDL/DCA-PEI/p53	Dichloroacetate	p53	Lung adenocarcinoma	Zhang et al. (2016a)
PLLA/PLGA	DOX	p53	Hepatocellular carcinoma	Xu et al. (2012, 2013)
Polyamidoamne dendrimer functionalized graphene oxide	DOX	MMP-9 shRNA	Breast cancer	Guo et al. (2017)
OEI600-PBA/HBPO	DOX	Beclin1 siRNA		Jia et al. (2015)
FA/PEG/liposomes (EPC, CHOL and DOTAP)	PTX	Tariquidar	Ovarian cancer	Sriraman et al. (2015)
PLGA	DTX	Gambogic acid	Breast cancer	Xu et al. (2016)
mPEG-g-PLL-b-Phe	DOX	P-gp siRNA	Breast adenocarcinoma	Suo et al. (2016)
Nanoscale metal-organic frameworks	Cisplatin prodrug	MDR gene-silencing siRNAs(Bcl-2, P-gp, and survivin)	Ovarian cancer	He et al. (2014)
Folate acid conjugated graphene oxide	DOX	Gold NPs		Chauhan et al., (2016)
PLGA	DTX	Gold NPs		Hao et al. (2015)
Metallo-supramolecular nanogel	DOX	Tetraphenylporphyrin zinc		Yao et al. (2015)
Mesoporous silica	DOX	Gold nanorods	Lung carcinoma	Chen et al. (2016)
Liposomes (EPC, CHOL, DOTAP, PEG2kPE)	PTX	Tariquidar	Ovarian cancer	Patel et al. (2011)
Transferrin conjugated Liposomes	DOX	VER	Leukemia	Wu et al. (2007)
FA-PEG-PLGA	cis-diaminodichloroplatinum	PTX	Non-small lung cancer	He et al. (2015)
β-CD modified CdSe/ZnSe QDs coupled to L-Arg or L-His	DOX	siRNA targeting the MDR1 gene	Cervical cancer	Li et al. (2012)
Gold nanorod	DOX	siRNA against ASCL1	Neuroendocrine carcinoma	Xiao et al., (2012a,b)
poly(styrene-co-maleic anhydride) derivative ith adipic dihydrazide	DOX	Disulfiram	Breast cancer	Duan et al. (2013)

Recently, various biomaterials are investigated for constructing therapeutic delivery carriers via tailoring their chemical and physical properties to meet specific needs in different situations. Polymers have been extensively studied for its potential to simultaneously deliver different chemotherapeutic agents with low aqueous solubility. Drugs with poor water-solubility are encapsulated with polymers to form micelle structures, where drugs reside within the hydrophobic core and hydrophilic chains form the shell of the micelle.

### 4.1. Liposomal materials

In the early 1960–1970s, Gregoriadis and Gregoriadis et al. first established the concept that liposomes could be used as drug carriers, just like ‘putting old drugs into new clothing’ (Gregoriadis & Ryman, 1960; Gregoriadis, 1973). Then in 1975, Kobayashi demonstrated that the liposomal cytarabine could significantly enhance the survival of leukemic mice *in vivo* compared to cytarabine alone (Kobayashi et al., 1975). Since the first description of liposomes as a vehicle for delivering cytostatic agents, liposomes gain a lot of attentions as NDDS and have been introduced in clinical applications. In 1995, pegylated liposomal doxorubicin (Doxil^®^ or Caelyx^®^) was the first nanocarrier approved by the FDA. Today, many delivery systems on the market or in clinical trials are liposomal or lipid-based products. Among them, two sets of liposomal formulation of small molecules drug combinations have entered clinical trials, which is CPX-351 (cytarabine/daunorubicin) in patients with acute myeloid leukemia, and CPX-1 (irinotecan/floxuridine) in patients with colorectal cancer (Weissig et al., 2014).

Liposomes are usually composed of phosphatidylcholine, either the natural mixtures obtained from soybean or egg yolk, or their hydrogenated derivatives, exhibiting a higher phase transition temperature. Hydrophilic drugs can be easily dissolved in the external aqueous core during liposome preparation using the common thin layer hydration method, while hydrophobic drug can dissolve between the lipid bilayers oil phase (Eloy et al., 2014).

Lipid nanocarriers tend to passively accumulate in tumor tissue via the enhanced permeability and retention (EPR) effect caused by the unique anatomical and pathophysiological characteristic of tumor vessels. Furthermore, PEGylation of the liposomal carriers can increase the circulation time of liposomes and consequently enhance their accumulation in tumor tissue, while reduce mononuclear phagocyte system uptake. Patel *et al* prepared stealth liposomes encapsulated with P-gp inhibitor tariquidar and drug PTX by thin film hydration method (Patel et al., 2011). A simultaneous delivery of tariquidar and PTX by long-circulating liposomes resulted in greater cytotoxicity in SKOV-3TR cells than paclitaxel alone, which demonstrated a significant reversal of the MDR towards PTX. Moreover, by synthetic modifying liposome surface or terminal PEG molecule with tumor specific ligands, such as folic acid, transferrin, or monoclonal antibodies, liposomes can be actively directed for recognition by receptors at the pathological site and consequently achieve tumor-targeted delivery. For instance, by polycarbonate membrane extrusion and transferrin (Tf) conjugation, Wu *et al* synthesized transferrin-conjugated liposomes (Tf-L-DOX/VER) co-encapsulated with DOX and verapamil (VER) (Wu et al., 2007). In DOX-resistant K562 cells, Tf-L-DOX/VER showed greater cytotoxicity than either non-targeted liposomes (L-DOX/VER) or Tf-targeted liposomes loaded with DOX alone (Tf-L-DOX), which indicated that this Tf receptor targeting co-delivery nanoformulation was highly effective in overcoming drug resistance.

Also, liposomes can be designed to respond to specific signals, such as hyperthermia, pH decrease, an alternation of external magnetic field or ultra-sound, to release their aqueous content. The triggered release was utilized to avoid unfavorable side effect in non-targeted sites and gain therapeutic efficacy enhancement.

### 4.2. Polymer-based materials

Polymeric nanoparticles are often considered as alternatives to liposomal vehicles for their improved *in vivo* stability and loading efficiency. Having polymer filled core, a variety of polymers have been studied and developed for drug delivery. Among them, poly (lactic acid) (PLA), poly(glycol acid) (PGA) and their copolymers, PLGA are most widely used for delivery systems development because of their outstanding biodegradability, biocompatibility, and ease of processing.

The conventional preparation of polymeric nanoparticles is mainly through dispersion of preformed polymers or polymerization of monomers. Most of these nanoparticles are formulated through a self-assembly process using block-copolymers consisting of two or more polymer chains with different hydrophilicity. These copolymers can spontaneously assemble into a core-shell structure in an aqueous environment. The core of these polymeric micelles plays the major role in dictating the nature of the molecules to be encapsulated. Hydrophobic polymers provide good microenvironments for solubilization of poorly water-soluble drugs. Furthermore, various types of polymeric blocks such as dendrimer, brush hyperbranched, block copolymer can be used to modify or supramolecular-assemble into polymeric nanoparticles by core- or shell-crosslinking or ligand modification.

Among various therapeutic polymeric materials, PLGA is one of the most successful ones used in clinics, owing to that the two endogenous monomers, which can be easily metabolized via the Krebs cycle and it has a minimal systemic toxicity. Now, PLGA is approved by the US FDA and European Medicine Agency (EMA) in various drug delivery systems for human use (Danhier et al., 2012).

Just like the liposomes described in last section, for the purpose to gain anti-cancer efficiency and to reduce unwanted side effect, there were also various ways developed to modify PLGA-based delivery system. For instance, Danhier’s group fabricated folic acid modified poly(ethylene glycol)-PLGA (FA-PEG-PLGA) nanoparticles to deliver CDDP and PTX together for non-small cell lung cancer (Danhier et al., 2012). The co-delivery formulation exhibits more effective antitumor effects than free drugs or single-drug-loaded nanoparticles by inducing apoptosis and cell cycle retardation. Xenograft lung cancer growth suppression was also found in xenograft mice, as well as prolonged survival time.

### 4.3. Inorganic materials

Increasing the use of inorganic material based nanoparticles, including gold nanoparticles, quantum dots and silica based nanoparticles, etc., in biological applications has been observed due to their tunable specific properties (Li et al., 2012; Pan et al., 2013).

One of the most studied nanotechnology-based inorganic delivery vesicles is gold nanoparticles. For the gold nanoparticles, they have shown great promise for cancer therapy in several applications, such as photo-thermal agents, radio-sensitizers, and potential drug carriers (Huang et al., 2010). Xiao et al. developed and characterized a pH-sensitive multifunctional gold (Au) nanorod (NR)-based nanocarrier for co-administration of doxorubicin and small interfering RNA (siRNA) against achaete-scute complex-like 1 (ASCL1) specifically to neuroendocrine cancer cells (Xiao et al., 2012b). This Au-DOX-OCT complexed with ASCL1 siRNA exhibited significantly higher gene silencing in NE cancer cells than non-targeted Au-DOX complexed with ASCL1 siRNA, as well as enhanced anti-proliferative effect. Thus, this tumor-targeting Au NR-based nanocarriers for combined chemotherapy and siRNA-mediated gene silencing holds significant potential in enhancing the therapeutic outcomes in treating neuroendocrine cancers.

MSNs are another important inorganic material that has been used for co-delivery. Their high-surface area to volume ratio and large pore volume make them ideal for loading large amounts of drugs and conjugation/complexation of other components on the surface. Shen et al. (2013) developed DOX-loaded mesoporous silica-encapsulated gold nanorods (GNRs@mSiO_2_), which were capable of combining photothermal therapy and chemotherapy. The smart co-delivery system enhanced cell-killing effect when A549 cells were exposed to both DOX-loaded GNRs@mSiO_2_ and near-infrared (NIR) illumination. Compared with chemotherapy or photo-thermal treatment alone, the combined treatment showed higher therapeutic efficacy and lower systematic toxicity *in vivo*.

Although inorganic nanoparticles attracted extensive attentions as promising delivery vesicles, only NanoTherm^®^ has been approved by FDA as drug delivery system in local ablation in glioblastoma, prostate, and pancreatic cancer. To date, none of inorganic NPs for co-delivery has reached market or get approved yet. Some of them are in early clinical testing, for example pegylated colloidal gold-TNFα particles for cancer therapy and silicon nanocarriers for parenteral peptide delivery.

### 4.4. Other materials

There are other nanoscale platforms that have been demonstrated to deliver multiple therapeutic cargoes in different combination, such as drug-conjugated nanoparticles and protein-base nanoparticles.

Duan et al. (2013) prepared a pH-sensitive polymeric micelles system by conjugating doxorubicin to poly(styrene-co-maleic anhydride) (SMA) derived with adipic dihydrazide (ADH) through a acid-cleavable hydrazone bond, and then encapsulated disulfiram (DSF) (a P-gp inhibitor and an apoptosis inducer) into the micelles. Compared with other combinations of both drugs, the formed SMA-ADH-DOX (SAD) conjugation had effectively increased intracellular accumulation of DOX, enhanced cytotoxicity and promoted apoptotic response, as well as the most effective inhibition on the growth of drug-resistant breast cancer xenografts.

Protein-base nanoparticles have been extensively studied as a delivery vesicle for combined anticancer therapy. For example, Li et al. (2015b) synthesized di-block copolypetides as a pH-sensitive drug delivery vector for delivery of DOX and siRNA. This novel pH-responsible polymeric vesicles can self-assemble into stable vesicles at neutral pH and disassemble in acid pH, such as in endosomal or lysosomal. The uptake assay showed that with the help of the vesicles, DOX and siRNA could accumulate in the same cells. So far, only one protein-base formulation has found its way into the clinic: albumin-nanoparticle-bound PTX (Abraxane®) is used for the therapy of breast cancer, non-small-cell lung cancer and pancreatic cancer.

## 5. Challenges and strategies

Nanotechnology has shown to be extremely useful in the co-delivery of genes, proteins and chemotherapeutic agents. NDCDS synergistically increases the therapeutic efficacies by inhibiting growth of the cancer tissue and minimizing the toxicity-side effects compared with free anticancer drugs. However, most of present studies about NDCDS are only focused on preparation methods, physicochemical characterization, release property of loaded-drugs in simulant microenvironment of cancer tissue, and *in vitro* or/and *in vivo* anticancer effects. Because pharmacological targets of some anticancer drugs locate at the cytoplasm or the nucleus of cancer cells, it is imperfect that NDCDS only carries these drugs to the domain of cancer tissue or the cytoplasm. Intracellular target delivery and precise release of the loaded-drugs should be an ultimate goal of NDCDS. Some extracellular and intracellular barriers will affect the realization of the goal.

**Figure 1.**
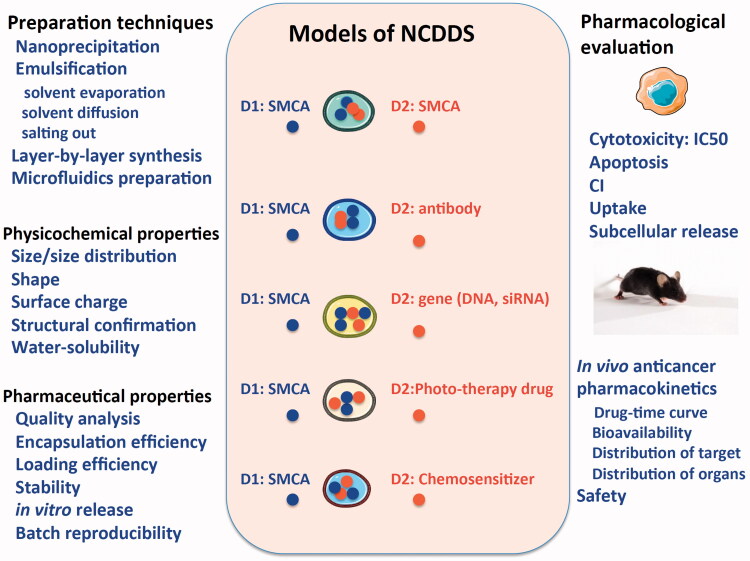
Schematic illustration of NDCDS models: the physicochemical and pharmaceutical properties, pharmacodynamic, and pharmacokinetic profiles.

The purpose of drug research is to market for clinical use. Thousands of publications suggested that NDCDS therapeutics exhibit effective *in vitro* or/and *in vivo* anticancer efficacy. However, there is no NDCDS successfully entering clinical trials, indicating the manufacturing of NDCDS for commercialization still has a long way to go. Some challenges affect the realization of the goal, for example, design and preparation techniques, the physicochemical and pharmaceutical property, and pharmacological and toxicity of NDCDS (Figure 1). Beside, for NDCDS, how to break biological barrier from the site of administration to pharmacological target is another challenge. These challenges will drive researchers to investigate strategies to optimize NDCDS for combination chemotherapy in future clinical trial.

### 5.1. Design and preparation techniques of co-delivery nanoparticles

To obstain a successful NDCDS, design needs to be considered at least in two aspects. One is the drug delivery material and another is the model drug. Excellent drug delivery material should have some characteristics, such as, biocompatibility or low toxicity, easy access, convenient preparation and so on. The high costs of the raw drug delivery materials and the need for a tedious multistep production process make the production of NDCDS therapeutics expensive. Even though, the existing drug delivery materials, such as PLGA, TPGS, and CHI, excellent performance no matter in research or in clinic. But they still have some inherent shortcomings. Therefore, the synthesis of its derivatives has become a research hotspot. A definite biological activity of the drug delivery material, such as TPGS, can affect the function of P-gp, and help to deliver anti-cancer drugs belonging to P-gp substrates. The researchers have been exploring the R&D of novel drug delivery materials.

At present, NDCDS preparation techniques include nanoprecipitation, emulsification-solvent evaporation, emulsification-solvent diffusion, emulsification salting out, layer-by-layer synthesis, microfluidics method, and so on. The co-delivery nanoparticles preparation techniques are at least mature at the laboratory phase. The amount of nano-products obtained in test tube is often at milligrams level, which can only be used for physicochemical characterization and cytological evaluation *in vitro*, and for *in vivo* animal testing with a slight amount of deficiency. From the perspective of preparation in laboratory phase, another noteworthy problem is that despite the advantage of easy scale-up of conventional techniques, the quality of samples obtained from different batches of preparation is not consistent.

Due to the fact that at least two drugs are loaded on NDCDS, the procedure for drug loading is also requires careful consideration in preparation. For example, when a small molecule drug is designed to be administered with protein, or gene, which is first loaded, the encapsulation efficiency and loaded drug rate will be significantly affected.

### 5.2. Physicochemical characteristics

Upon completion of the preparation of NDCDS, its characterization is an essential test. In general, the characterization of nanoparticles includes particle size and size distribution, commonly used electron microscope observation and DLS detection, appearance shape, surface charge (Zeta potential), water solubility, and so on. It is also necessary to provide evidence that the drug and the material are physically mixed or chemically cross-linked by FT-IR, mass spectrometry (MS), or X-diffraction. Therefore, NDCDS should be characterized using multiple methods. However, the real problem is that these physicochemical characteristics are not sufficient to indicate the state of the nanoparticles in practical applications. For example, NDCDS will be formulated in aqueous in future animal test or human clinical treatment. In this condition, NDCDS may tend to aggregation or agglomeration as a result of interaction with some biological fluids (blood, or tissue fluid) or biomolecules (albumin, or fibrinogen). In practical applications, even small variations of physicochemical characteristics maybe bring dramatic changes to *in vivo* anticancer effect and toxicity. Therefore, the physicochemical characterization of NDCDS should be checked under simulated physiological or pathologic conditions, instead of only in the condition of freeze-dried powder or in buffer.

The surface charge is one of the most important properties of nanoparticles, which affects the ability of their uptake by the cells. Nanoparticles with positive charge can escape from endo-lysosome more easily while negative charged ones are beneficial for transportation in vessel. In recent years there are many researches committed to study the surface charge of nanoparticles, such as charge reversal materials like PLGA and some inorganic materials. In 1986, EPR effect was proposed as enhanced permeability and retention effect of solid tumors, that is, compared with normal tissue; some molecules or particles of special size tend to accumulate in tumor tissue. EPR effect promotes the selective distribution of macromolecular substances in tumor tissue, which subsequently increase the treatment efficacy and reduce the side effects of the system. However, it is worth noting that the EPR effect can only make nanoparticles passively distributed around tumor tissue, not delivered into cells and release chemotherapeutic drugs to play a role in cancer prevention. For most anticancer drugs, their target spot is cytoplasm or nuclei, which makes efficacy exertion of these drugs potentially challenging. Therefore, the precise release of drugs in nanoparticles in the target site is the premise to play the anti-cancer efficacy.

### 5.3. Pharmaceutical characteristics

Although NDCDS is relied on the concept of combination chemotherapy, there are slightly differences between each other. For example, in clinical practice, the drugs for combination chemotherapy are administered rarely mixing in the same infusion bottle, while NDCDS make the loaded drugs enter the blood circulation at the same time. This fact poses a challenge to pharmaceutical characteristics.

First of all, it remains challenging that multiple drugs with different physicochemical properties, for example molecular weight, solubility, and p*K*a, are loaded in similar NDCDS. These drugs need to have precise molar ratios. Precise control over loading efficiency of co-delivery drugs would enable dosage optimization and synergism maximization among the encapsulated compounds. However, they are often difficult to control, especially macromolecular drugs, antibodies, proteins, DNA or RNA.

Aside from that, sequential and precise drug release is another impactful parameter of determining the synergistic action of co-delivery drugs. However, many studies had carried out to investigate on the drug release of NDCDS in model microenvironment with pH buffer of normal body fluid, or tumor tissue and cell. In addition, it is sometimes difficult to show the real extent of drug release. In fact, how to confirm the in situ distribution of loaded drugs in the cell and tissue *in vivo* is more meaningful than in the *in vitro* simulation test. However, NDCDS makes drugs enter the blood circulation at the same time. Hence, it is a feasible approach for cancer chemotherapy to adopt an excellent NDCDS with a sequential and precision release characteristic to transport anticancer drugs to their pharmacological targets (He et al., 2015).

One last thing, stability characterization of NDCDS based on storage aspects is also challenging. Because long-term storage in aqueous solutions may alters the properties of the nanoparticles. So, NDCDS should be stored in a lyophilized powder form. While NDCDS is used *in vivo* animal test or clinical human treatment, it must be re-suspended in saline or 5% glucose injection. In this condition or after reaching biological fluids such as blood, NDCDS can change their physicochemical properties again, such as size, surface charge, and release profile, which may have an impact on their performance *in vivo*.

### 5.4. Pharmacological property of the model drugs

For NDCDS, the choice of drug delivery material is the first issue, and at the same time the determination of the model drug is equally important (Hapala, 1997). As mentioned above, co-delivery is based on the concept of clinical combined chemotherapy. It also should follow the principle of combination chemotherapy. Therefore, the fundamental choice of model drugs is better to have clinical evidence. Examples of combination chemotherapies used in clinic include carboplatin and PTX for treatment of ovarian cancer. Secondly, the selected drugs should have obvious shortcomings in clinical application. For instance, most chemotherapeutic drugs are hydrophobic cytotoxic small molecules, and common problems include insolubility, poor bioavailability, and lack of cellular specificity, serious systemic toxicity, and the development of MDR.

In addition, it is necessary to consider whether some of the pro-drugs are essential for nanoparticle delivery. Irinotecan is metabolized to 7-ethyl-10-hydroxycamptothecin (SN-38) via hydrolysis by carboxylesterases and via glucuronidation by UGT1A1 in the liver. SN-38 inhibits both replication and transcription of DNA via active the topoisomerase I, which have 1000 times more activity than its parent irinotecan. So, it is not an advisable choice for irinotecan to be loaded in NDCDS.

**Figure 2.**
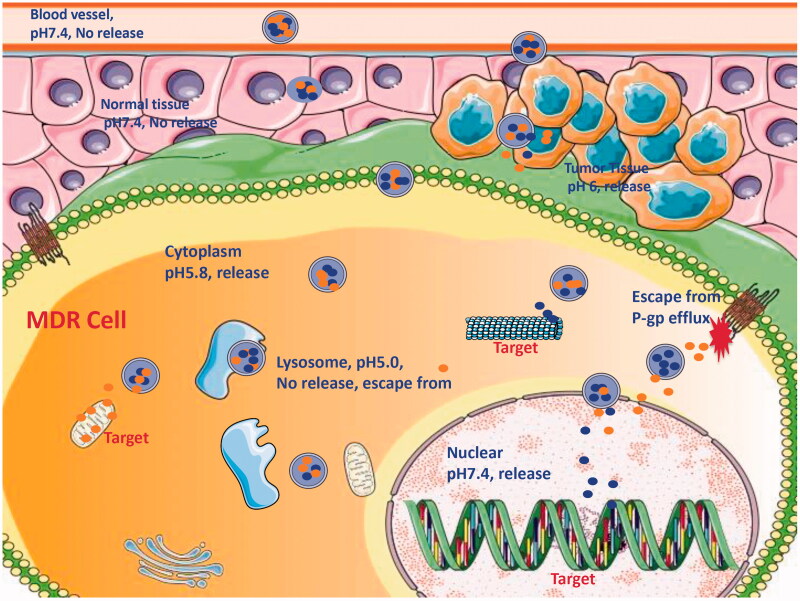
Mechanisms illustration of NDCDS reversing MDR: the potential process of from blood to pharmacological target.

Because of pharmacological targets of some anticancer drugs locating at the cytoplasm or the nucleus of MDR cell, it is insufficient that NDCDS only carries these drugs to the domain of cancer tissue or the cytoplasm. Some intracellular barriers, for example, the efflux associated with P-gp, the detention from endosome or lysosome, and the obstruction of the nuclear envelope will hamper the delivery of loaded drugs to the target (Figure 2). Loaded drugs in NDCDS using separate carriers have benefits with characteristic of controlling drug release rate and the domain. Meanwhile, it is generally considered that NDCDS deliver drugs to pharmacological targets by targeting, anchoring, uptake, intracellular migration, localization, and into the nucleus. To study visually the process, it needs to prepare the fluorescently labeled nanoparticles.

### 5.5. Safety

The safety of NDCDS largely depends on the materials for drug delivery and model drugs. In general, the materials for drug delivery are mostly non-toxic or low toxic; but they may have some biological effects when transformed to nanoscale. At the cellular level, it has been reported that nanoparticles can cause inflammation, oxidative stress, or mitochondrial dis-function (Kemp et al., 2015).

The major contributors to the toxicity of NDCDS come from co-delivery anticancer drugs, particularly small molecule cytotoxic drugs. One of the principles of combined chemotherapy is that toxicity of drugs does not overlap. Nevertheless, many small molecule chemotherapy drugs induce similar toxicity or side effects, for instance, bone marrow suppression causing anemia and leukopenia, lipsotrichia, immunosuppression, and gastrointestinal mucosal damage, etc.

Currently, the studies on the toxicity of NDCDS were restricted to IC_50_ at cell level, or *in vivo* acute test on animal model. In fact, analyzing chronic toxicity is more important, yet data on this subject is extremely scarce. The reason may be related to experimental benefit–cost ratio; in addition, the tested sample of NDCDS had some difficulties to provide for long-term animal test. As a consequence, how to provide adequate uniform quality of the test sample is an urgent problem to be solved. Another worth considering is that the free drug should be set as the control group to compare with the toxicity of its nano-formulation in animal experiment.

### 5.6. Biological barrier from the site of administration to the pharmacological target

A large number of anticancer drugs target the nucleus, which bind with nuclear DNA or associated with enzymes to exert their cytotoxicity to cancer cells. One of the obstacles on NDCDS for combination chemotherapy is how to deliver different drugs to the same target sites.

Taking brain tumor chemotherapy as an example, we try to describe the biological barrier from the site of administration to the pharmacological target. The drugs for glioblastoma, also known as glioblastoma multiforme (GBM), include temozolomide, bevacizumab, DOX, and teniposide. At first, the central nervous system (CNS) represents a formidable challenge for the delivery of these therapeutic agents due to the blood–brain barrier (BBB). This physical barrier limits the brain uptake of the vast majority of neurotherapeutics agents. Then, the cytomembrane of cancer cell constitutes the second barrier by hindering the entry of anticancer drug into the cell interior. After anticancer drug being carried to cytoplasm, it is worth mentioning that the chemotherapeutic drug can be pump out. Also, the escape of NDCDS from the degradation of endo-lysosomal vesicles is another challenge. At last, the nucleus targeting anticancer drugs need to be further delivered to the nucleus for actions; the nuclear envelope is also the obstruction. In brief, we believe that continuous research efforts on NDCDS will provide some solutions to breaking the biological barrier to achieve the goal of effective cancer treatment (Hu et al., 2016).

Pharmacokinetics describes the action of the body over drug, including absorption, distribution, metabolism and excretion (ADME). For NDCDS, ADME is also of great significance for the study of the process of loaded drug *in vivo*. Some published articles on NDCDS had studied the pharmacokinetic parameters and bioavailability of loaded drug. It is worth noting that the distribution of drugs in the pharmacological targets, including cancer tissue, cell, and subcellular structure, is more important than the parameters obtained from the drug–time curve of plasma. Yet, data are in urgent need.

On the one hand, the simultaneous detection of loaded drugs in the NDCDS requires more specific and more sensitive analysis methods; on the other hand, the drugs released in the target maybe have an effect on each other. For chemotherapy drugs, *in situ* detection of the target is also a challenge. If the drug does not emit fluorescence, it is necessary to use other fluorescent probes as an alternative to speculate the target of the drug delivery. For macromolecular drugs, it is more difficult to determine whether they have been delivered to the target area (Nastiuk & Krolewski, 2016; Teo et al., 2016).

Some analysis methods, such as HPLC, HPLC/MS, fluorescence microscopy, and *in vivo* animal imaging, have been used for the detection of loaded drugs in NDCDS of *in vitro and in vivo* biological specimen. Each method has its own advantages and shortcomings. Only by combining these methods, the results of the experiments can be used to determine the distribution of loaded drugs in the target. In summary, sufficient data have proven that NDCDS has broken through numerous biological barriers and delivered the loaded drugs to their final pharmacological target (Gilmore et al., 2015).

## 6. Conclusion

To achieve superior therapeutic efficacy, the combination chemotherapy using two or more anticancer drugs in clinical practice has been widely accepted as a feasible strategy. On account of the concept of combination chemotherapy, co-delivery of anticancer drugs with nanotechnology gradually becomes a desired strategy and one of the research frontiers on innovative drug delivery. In recent years, NDCDS, which loads at least two anticancer drugs with different physicochemical and pharmacological properties into a combination delivery system, has achieved rapid development. NDCDS synergistically inhibited the growth of the tumor compared with the free drugs. In this review, we highlighted the current state of co-delivery nanoparticles and the most commonly used nanomaterial, discussed challenges and strategies, and prospective future development. Think that with the deepening of the study, the strategy of NDCDS will bring more and more inspirations for future cancer clinical treatment. We will eventually see the commercialization of NDCDS products for clinical treatment of cancer in order to prolong the life, or to improve life quality of patients.
